# Male Sex Is an Associated Factor for Delayed Bone Union Following Hybrid Closed-Wedge High Tibial Osteotomy

**DOI:** 10.3390/medicina60111772

**Published:** 2024-10-29

**Authors:** Naoya Kikuchi, Norihito Arai, Kosuke Okuno, Akihiro Kanamori

**Affiliations:** 1 Department of Orthopaedic Surgery, Institute of Medicine, University of Tsukuba, 1-1-1 Tennodai, Tsukuba 305-8575, Ibaraki, Japan; 2 Department of Orthopaedic Surgery, Tsukuba Memorial Hospital, 1187-2 Kaname, Tsukuba 300-2622, Ibaraki, Japan

**Keywords:** hybrid closed-wedge high tibial osteotomy, bone union, knee osteoarthritis, computed tomography

## Abstract

*Background and Objectives*: Although hybrid closed-wedge high tibial osteotomy (HTO) is an effective procedure for varus knee osteoarthritis, delayed bone union is a frequent occurrence. The rate of bone union and its associated factors remain unclear. This study aimed to investigate the detailed process of bone union in hybrid closed-wedge HTO using computed tomography (CT) and to examine factors associated with delayed bone union. *Materials and Methods*: We retrospectively reviewed 53 consecutive patients who underwent hybrid closed-wedge HTO. Cases with no bone union at any of five sites (anterior flange, posterior, lateral, hinge, and medial) on coronal and sagittal computed tomography at 3 months postoperatively were defined as delayed bone union. Regression analysis was performed with delayed bone union as the dependent variable and sex, age, height, body weight, body mass index, correction distance, smoking, and diabetes mellitus as independent variables. *Results*: Among 50 analyzed knees (mean age, 61.4 ± 7.8 years), 17 (34.0%) had delayed bone union at 3 months postoperatively and one knee had screw breakage. Sex was the only significant factor associated with delayed bone union (male: β = 2.1, *p* < 0.004). *Conclusions*: Delayed bone union (absence at 3 months after hybrid closed-wedge high tibial osteotomy) occurred in 34% of patients, and male sex was associated with delayed bone union.

## 1. Introduction

Good long-term results have been reported for medial open-wedge and lateral closed-wedge high tibial osteotomy (HTO) in varus knee osteoarthritis (OA) [1]. Hybrid closed-wedge HTO is a surgical technique to reduce patellofemoral pressure that cannot be addressed by open-wedge HTO [2,3,4,5]. 

Hybrid closed-wedge HTO is a surgical technique that creates a hinge point at three-quarters of the osteotomy line from the lateral side, closes the lateral side, and opens the medial side. Although hybrid closed-wedge HTO has advantages such as less bone resection, and by opening the medial side the superficial medial collateral ligament becomes tenser and the joint becomes stable, a previous study reported that it took an average of 4.5 months for bone union in hybrid closed-wedge HTO, with 25% of cases taking more than 6 months, as assessed by radiography [6].

Risk factors for delayed bone union or nonunion after open-wedge HTO, such as smoking, obesity, large opening gaps, unstable hinge fractures, and plate positioning, have been reported [7,8,9,10]. However, to the best of our knowledge, few studies have specifically focused on factors associated with delayed union in hybrid closed-wedge HTO. Hybrid closed-wedge HTO is a “no medial cortical hinge” procedure that requires an appropriate postoperative rehabilitation program. Understanding the bone union process and factors associated with delayed bone union is essential. However, the detailed process of bone union has not been fully elucidated, and factors associated with delayed bone union remain unclear. Male sex, previously identified as a risk factor for nonunion in tibial fractures [11], may similarly play a role in delayed union following hybrid closed-wedge HTO.

The purpose of this retrospective study was to investigate the detailed process of bone union in hybrid closed-wedge HTO using computed tomography (CT) and to examine the factors associated with delayed bone union. We defined delayed bone union as the absence of bone union at 3 months postoperatively because radiological parameters at 3 months postoperatively are associated with long-term nonunion in tibial fractures [12].

## 2. Materials and Methods

### 2.1. Participants

This study was approved by the Medical Ethics Committee of our institute and was conducted in accordance with the Declaration of Helsinki. The requirement for informed consent was waived because of the retrospective study design.

All patients who underwent hybrid closed-wedge HTO for varus knee osteoarthritis performed by the one knee surgeon with over 20 years of experience between March 2019 and March 2022 were considered for inclusion in this study. Patients with symptomatic unicompartmental OA of the medial femorotibial joint were included in the study. Hybrid CWHTO was performed when the planned correction distance exceeded 12 mm. It was also indicated in patients with a correction distance of less than 12 mm if the severity of patellofemoral OA, as seen on radiographs, was greater than stage III. Patients who could not be followed up with or who did not undergo postoperative radiological evaluation were excluded. Information on patient characteristics, including sex, age at the time of surgery, height, body weight, body mass index, smoking status, and diabetes mellitus, was obtained from medical records. Surgical findings, specifically correction distance, were recorded. In addition, the occurrence of reoperation was investigated.

### 2.2. Surgical Procedure and Postoperative Rehabilitation

Hybrid closed-wedge HTO was performed according to the method described by Takeuchi et al. [3]. Preoperative planning was performed using full-limb standing radiography. The mechanical axis was determined by drawing a line from the center of the femoral head to the center of the tibial plafond. The ratio of the distance between the point where the mechanical axis passed and the medial edge of the tibia to the distance of the width of the tibial plateau was defined as the mechanical axis percentage (%MA) [13]. The target %MA for surgical planning was 62.5% [14].

A pneumatic thigh tourniquet was applied to the operative leg. Arthroscopic examination was performed to verify the status of the articular cartilage and menisci before HTO in all cases. After arthroscopy, fibular osteotomy was performed, removing approximately 10–20 mm in length of bone to allow for tibial correction through a separate incision. Subsequently, tibial osteotomy was performed. The hinge point was set at three-quarters of the osteotomy line from the lateral side, dividing the proximal tibial osteotomy line by approximately 1:3. Osteotomy was performed along the proximal and distal osteotomy lines, from the proximal lateral cortex to the hinge point, using a bone saw and chisel. The length of the resected lateral tibial cortex was measured with calipers and defined as the correction distance. A separate ascending biplanar osteotomy cut was made behind the patellar tendon insertion in the frontal plane, retaining the tibial tuberosity at a thickness of approximately 10 mm. After removing the lateral closed-wedge bone block, the medial cortex was cut according to the proximal osteotomy line. Finally, the medial side was opened, the lateral side was closed, and plate fixation was performed using a TriS locking plate (Olympus Terumo Biomaterials, Tokyo, Japan).

All patients underwent the same rehabilitation protocol. Joint range of motion exercises were started on the day after surgery. One-third partial weight-bearing was started at 3 weeks postoperatively, one-half partial weight-bearing was started at 6 weeks postoperatively, two-thirds of partial weight-bearing was started at 9 weeks postoperatively, and full weight-bearing was started at 3 months postoperatively. Thereafter, the patient was instructed to walk with a T-cane if pain persisted while walking.

### 2.3. Radiographic and Clinical Evaluations

%MA was measured preoperatively and at 1 year postoperatively on full-length standing lower extremity radiographs. The medial proximal tibial angle (MPTA) [15] was measured in the anteroposterior view of the knee preoperatively and at 1 week and 1 year postoperatively. The posterior tibial slope (PTS) [16,17] was measured in the lateral view of the knee preoperatively and at 1 week and 1 year postoperatively.

**Figure 1:**
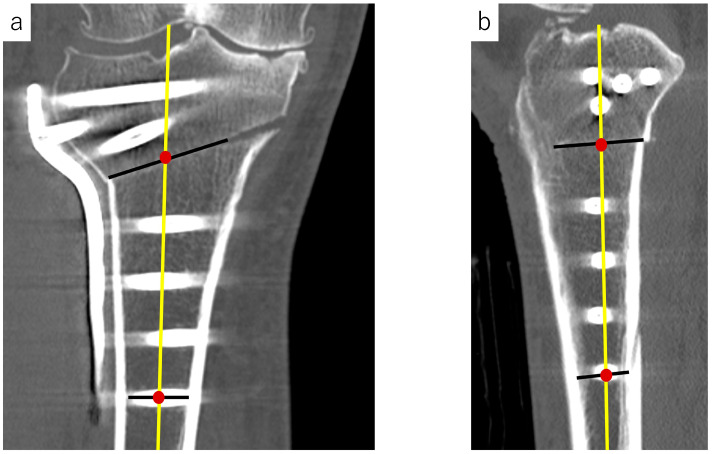
Definition of coronal and sagittal plane slices. (**a**) Coronal slices were defined as slices connecting the midpoint of the osteotomy line and the midpoint of the trabecular bone width at the height of the most distal screw in sagittal slices (yellow line on (**b**)). (**b**) Sagittal slices were defined as slices connecting the midpoint of the osteotomy line and the midpoint of the trabecular bone width at the height of the most distal screw in coronal slices (yellow line on (**a**)).

**Figure 2:**
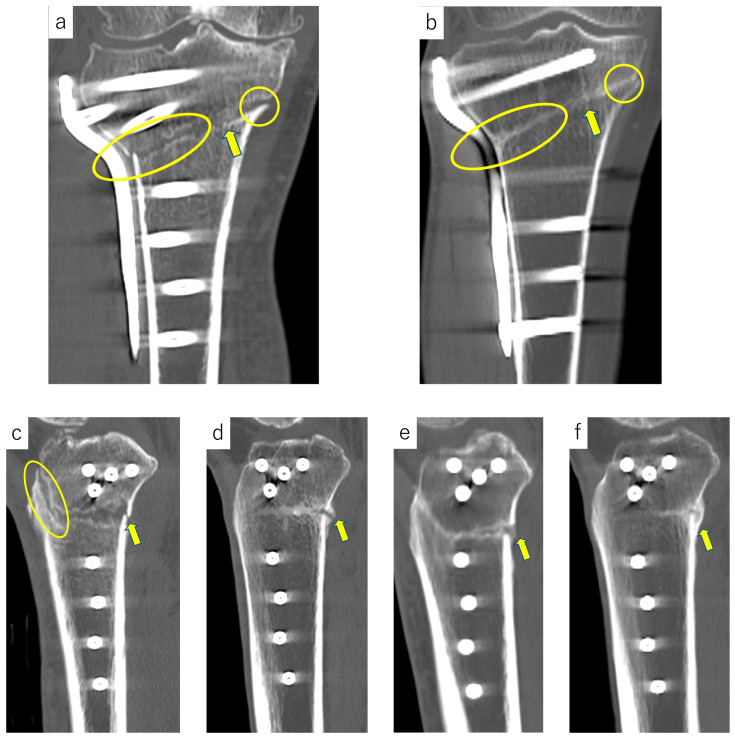
Evaluation of bone union. (**a**) Coronal slice at 3 months postoperatively. There is no callus at the lateral site (ellipse), hinge point (arrow), and medial site (circle). (**b**) Coronal slice at 3 months postoperatively. There is continuous remodeled callus at the lateral site (ellipse), hinge point (arrow), and medial site (circle). (**c**) Sagittal slice at 3 months postoperatively. There is no callus at the anterior flange (ellipse) and posterior site (arrow). (**d**) Sagittal slice at 3 months postoperatively. There is discontinuous callus at the posterior site (arrow). (**e**) Sagittal slice at 3 months postoperatively. There is continuous bridging immature callus at the posterior site (arrow). (**f**) Sagittal slice at 3 months postoperatively. There is continuous remodeled callus at the posterior site (arrow).

CT images obtained at 3 months, 6 months, and 1 year postoperatively were evaluated to assess bone union using Osirix imaging software (Pixmeo, Geneva, Switzerland). Coronal and sagittal slices were defined as those connecting the midpoint of the respective osteotomy line to the midpoint of the trabecular bone width at the height of the most distal screw (Figure 1). Medial, hinge, and lateral sites were evaluated using coronal images, whereas the anterior flange and posterior sites were evaluated using sagittal images. Bone union was investigated using the Tomographic Union Score, as previously described [18]. Based on this score, the callus was classified into the following four categories: no callus (Figure 2a,c), discontinuous callus (Figure 2d), continuous bridging immature callus (Figure 2e), and continuous remodeled callus (Figure 2b,f). Continuous bridging immature callus and continuous remodeled callus were classified as bone union [19]. The absence of callus or discontinuous callus at any of the above five sites at three months postoperatively was defined as delayed bone union.

Two orthopedic surgeons (N. K. and N. A.) with specialized training assessed all radiographs on two separate occasions, with a time interval of 6 weeks between evaluations. The inter- and intra-observer reliabilities of the assessments were determined using the intraclass correlation coefficient, and the 95% confidence intervals for both inter- and intra-observer reliabilities exceeded 0.85.

As a clinical evaluation, the Japanese Orthopedic Association (JOA) score was assessed preoperatively and at 1 year postoperatively [20].

### 2.4. Statistical Analysis

Continuous variables are presented as the mean ± standard deviation. The normality of data distribution was assessed using the Shapiro–Wilk test. For continuous variables, the paired *t*-test and Wilcoxon signed-rank test were used to compare variables before and after surgery when parametric assumptions were met and not met, respectively; the Pearson’s chi-squared test was used to compare qualitative variables. Group differences (delayed bone union group vs bone union group) were evaluated using the Student’s *t*-test for normally distributed variables, and the Mann–Whitney U test for non-normally distributed continuous variables. To investigate the factors associated with delayed bone union, a binomial logistic regression analysis was performed, with delayed bone union as the dependent variable and sex, age, height, body weight, body mass index, correction distance, smoking, and diabetes mellitus as independent variables. Statistical analyses were conducted using SPSS software (version 26.0; IBM Corp., Armonk, NY, USA). Statistical significance was defined as *p* < 0.05.

## 3. Results

Of 53 patients who underwent primary single-bundle anterior cruciate ligament reconstructive surgery during the study period, 3 were excluded because of loss to follow-up (*n* = 2) or missing CT evaluations (*n* = 1). Consequently, 50 patients were included in this study (Table 1). Among these, one patient (male, smoker) experienced screw breakage at 6 months postoperatively. The radiographic parameters of all patients, except one who underwent reoperation, are shown in Table 2. The JOA score improved significantly, from 67.6 ± 11.0 preoperatively to 87.7 ± 6.1 at 1 year postoperatively.

**Figure 3:**
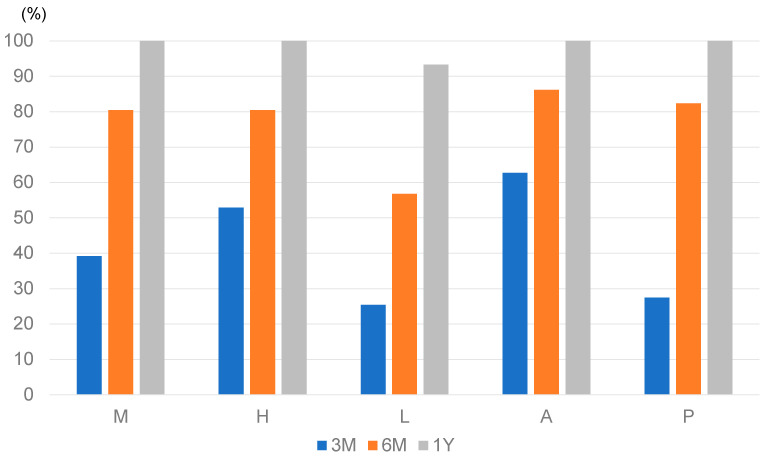
Bone union rate at each site on computed tomography at 3 months (3 M), 6 months (6 M), and 1 year (1 Y) postoperatively. M, medial site. H, hinge point. L, lateral site. A, anterior flange. P, posterior site.

The postoperative bone union rates at each site are shown in Figure 3. At 3 months postoperatively, bone union was most frequently observed at the anterior flange, and at 6 months postoperatively, 80% of all sites achieved bone union, with the exception of the lateral site. At 1 year postoperatively, bone union had occurred at all sites in all but three knees. At 3 months postoperatively, 17 knees had delayed bone union and 33 knees had union in at least one site.

The only statistically significant difference between the two groups was the proportion of male patients (Table 3). Further, binomial logistic regression analysis indicated that delayed bone union was associated with male sex (β = 2.1, *p* = 0.004) (Table 4).

## 4. Discussion

The most important finding of this study was that the rate of delayed bone union (defined as the absence of bone union at 3 months postoperatively) was 34% and male sex was the only factor associated with delayed bone union.

In a previous study on bone union in hybrid closed-wedge HTO, union of the osteotomy line was evaluated on anteroposterior knee radiography, and the union rate at 3 months postoperatively was approximately 20% [6,19], which is similar to the union rate at the lateral site on CT in the present study. We found that 34% of patients did not have union at all five sites at 3 months postoperatively, which is prior to planned full weight-bearing. For such patients, delaying full weight-bearing resulted in 92% union at 6 months postoperatively and 100% union at 1 year postoperatively, with the exception of one patient who had to undergo reoperation.

In the present study, the only factor associated with delayed bone union was male sex. Male sex has been reported as a risk factor for nonunion in tibial fractures [21]; however, the reason for more difficult bone union in male patients remains unclear. Possible sex-related differences include body weight and bone mineral density. Kawai et al. [22] reported heavier body weight and higher bone mineral density as associated with delayed bone union at 3 months after conventional closed-wedge HTO. Heavy body weight is detrimental to bone union because of the large mechanical load placed on the plate. Further, a previous report found that high bone mineral density was correlated with body weight [23]. Whether there is an association between bone mineral density and delayed bone union requires further investigation. Although mechanical instability may be the most likely cause, it is interesting to note that delayed bone union was more common in male patients, and this is an important topic for future research.

In our postoperative rehabilitation program, patients began partial weight-bearing at 3 weeks postoperatively and were allowed full weight-bearing at 3 months postoperatively; thus, our patients started weight-bearing later than those in previous reports [6,19]. Early postoperative weight-bearing in open-wedge HTO leads to better early clinical outcomes [24], and early weight-bearing may be desirable in hybrid closed-wedge HTO. Additionally, there have been reports of decreased pain as bone union progresses in hybrid closed-wedge HTO [6]. The insertion of additional screws has been shown to accelerate bone union [19], and stronger fixation through supplementary procedures could enable earlier weight-bearing and contribute to pain reduction. This is the first study to identify factors associated with delayed bone union in hybrid closed-wedge HTO. Based on our findings, supplementary procedures may benefit male patients. Further prospective studies are needed to validate these findings.

This study has several limitations. First, this was a retrospective study and a selection bias may have occurred. Second, the sample size was small. Therefore, there is a need for additional prospective studies assessing larger sample sizes. Third, clinical follow-up was limited to 1 year postoperatively. If clinical evaluations were conducted in the early postoperative period (e.g., at 3 or 6 months postoperatively), the disadvantages of delayed bone union may be more clearly observed, and the advantages of accelerated bone union may be demonstrated in more detail. Finally, we did not use patient-reported outcome measures in the clinical evaluation.

## 5. Conclusions

Bone union was not observed in 34% of patients at 3 months postoperatively following hybrid closed-wedge HTO, as assessed by CT scans. Male sex was associated with delayed bone union.

## Figures and Tables

**Table 1:** Patient demographic data.

Parameter	Total *n* = 50
Sex (male/female)	26/24
Age	61.4 ± 7.8
Height (m)	1.62 ± 0.07
Body weight (kg)	68.3 ± 11.9
BMI (kg/m^2^)	26.1 ± 3.6
Correction distance (mm)	9.9 ± 1.8
Smoking	5
Diabetes mellitus	2
Pre-%MA	9.6 ± 12.2
Pre-MPTA	83.2 ± 2.4
Pre-PTS	6.3 ± 2.8

BMI, body mass index; %MA, percentage of mechanical axis; MPTA, medial proximal tibial angle; PTS, posterior tibial slope.

**Table 2:** Radiological and clinical outcome changes before and after hybrid closed-wedge high tibial osteotomy.

Parameter	Preoperatively	1 Year Postoperatively	*p*-Value
%MA	9.4 ± 12.2	59.6 ± 9.2	<0.001
MPTA (degree)	83.2 ± 2.4	93.7 ± 2.4	<0.001
PTS (degree)	6.4 ± 2.8	3.4 ± 3.1	<0.001
JOA score	67.6 ± 11.0	87.7 ± 6.1	<0.001

%MA, percentage of mechanical axis; JOA, Japanese Orthopaedic Association; MPTA, medial proximal tibial angle; PTS, posterior tibial slope.

**Table 3:** Comparisons in characteristics between delayed bone union and bone union groups.

Parameter	Delayed Bone Union (*n* = 17)	Bone Union (*n* = 33)	*p*-Value
Sex (male/female)	14/3	12/21	0.002
Age (years)	62.2 ± 8.1	61.0 ± 7.7	0.53
Height (m)	1.65 ± 0.08	1.61 ± 0.07	0.05
Body weight (kg)	71.9 ± 11.1	66.9 ± 12.2	0.24
BMI (kg/m^2^)	26.5 ± 2.9	25.9 ± 3.9	0.53
Correction distance	10.4 ± 1.6	9.6 ± 1.9	0.25
Smoking	3	2	0.20
Diabetes mellitus	0	2	0.30

BMI, body mass index.

**Table 4:** Factors related to delayed bone union.

Parameter	β	*p*-Value
Male sex	2.1	0.004
Age (years)	0.03	0.43
Height (m)	0.06	0.13
Body weight (kg)	0.03	0.23
BMI (kg/m^2^)	0.03	0.72
Correction distance (mm)	0.23	0.20
Smoking	0.80	0.45
Diabetes mellitus	−20.5	0.99

BMI, body mass index.

## Data Availability

The original contributions presented in the study are included in the article/Supplementary Materials; further inquiries can be directed to the corresponding author.

## Supplementary Materials

The following supporting information can be downloaded at: https://www.mdpi.com/article/10.3390/medicina60111772/s1, Table S1: STROBE Statement.
